# Elevated serum IL-2 and Th17/Treg imbalance are associated with gout

**DOI:** 10.1007/s10238-023-01253-4

**Published:** 2024-01-19

**Authors:** Xiaoyu Zi, Ronghui Su, Rui Su, Hui Wang, Baochen Li, Chong Gao, Xiaofeng Li, Caihong Wang

**Affiliations:** 1 Department of Rheumatology, the Second Hospital of Shanxi Medical University, Taiyuan, Shanxi China; 2 Shanxi Key Laboratory of Immunomicroecology, Taiyuan, Shanxi China; 3 Pathology, Joint Program in Transfusion Medicine, Brigham and Women’s Hospital/Children’s Hospital, Harvard Medical School, Boston, MA USA

**Keywords:** Early-onset gout, Late-onset gout, T helper 17 cells, Regulatory T cells, IL-2

## Abstract

Gout is considered an auto-inflammatory disorder, and the immunological drivers have not been fully unraveled. This study compared the peripheral lymphocyte and CD4^+^T cell subsets, and cytokines in gout and healthy controls (HCs) to explore the contributions of T helper 17 (Th17) cells, T regulatory (Treg) cells and cytokines to the pathogenesis of gout. We enrolled 126 gout patients (53 early-onset gout with age of first presentation < 40 years, and 73 late-onset gout with age of first presentation ≥ 40 years) and 77 HCs. Percentage and absolute numbers of peripheral lymphocyte and CD4^+^T cell subpopulations in each group were detected by flow cytometry. The serum cytokine levels were determined by flow cytometric bead array. For circulating CD4^+^T cell subsets, Th17/Treg ratio was significantly higher in early-onset gout, late-onset gout and gout without tophus than HCs; Th17 cells were significantly elevated in early-onset gout and gout without tophus, while the percentage of Treg cells was significantly decreased in early-onset and late-onset gout. Additionally, gout patients had significantly higher cytokines levels (including IL-2, IL-4, IL-6, IL-10, IL-17, IFN-γ, and TNF-α) than HCs; IL-2 levels were positively correlated with Treg cells and negatively correlated with ESR. ROC analysis showed that disease duration, CRP and fibrinogen, had moderate predictive performances for tophus in gout (the AUCs were 0.753, 0.703 and 0.701, respectively). Our study suggests that early-onset and late-onset gout differ in Th17/Treg imbalance, which in early-onset gout is due to elevated Th17 cells and in late-onset gout is due to decreased Treg cells. And increased serum cytokine levels, especially IL-2, may play an essential role in that. Restoring Th17/Treg balance may be a crucial way to improve the prognosis of gout patients.

**Supplementary Information:**

The online version contains supplementary material available at 10.1007/s10238-023-01253-4.

## Introduction

Gout is one of the most common inflammatory joint diseases [1], and its prevalence and incidence have substantially increased over recent years [2]. As reported, the number of early-onset gout (defined as the development of symptoms before the age of 40) is increasing [3, 4]. In gout, monosodium urate (MSU) crystals are accumulated in and around the joint, which triggers a strong inflammatory response and excruciating pain [5, 6]. Recurrent gout attacks can lead to tophus formation, joint deformities and damages, and movement disorders [5, 7]. Furthermore, some studies suggest that other illnesses like hypertension, obesity, atherosclerotic cardiovascular disease (CVD), diabetes, dyslipidaemia, chronic kidney disease (CKD), and kidney stones are frequently linked to gout [5, 8, 9], all of which seriously harm people’s health. Thus, it is vital to investigate what causes gout to develop early-onset.

Gout was traditionally considered to be a classic inflammatory disease characterized by the excessive production of interleukin (IL)-1β and hyperactivity of innate immune cells [10, 11], in which neutrophils and macrophages are the main immune cells involved [12]. It has been discovered that MSU crystals activate infiltrating neutrophils by promoting neutrophil extracellular traps (NETs) formation in gout [13]. NETs release various danger-associated molecular patterns, such as histones or NLRP3 inflammasomes or Toll-like receptors [14], which can convert inactive IL-1β precursor to active IL-1β and significantly contribute to the quick generation of mature IL-1β and subsequent inflammatory response [15, 16]. Nevertheless, the exact pathogenesis of gout has not yet been completely elucidated and cannot be fully explained by the traditional theory. It has been reported that gout is related to metabolic syndrome, renal diseases, and cardiovascular disease [17]. Importantly, the prominent role of CD4^+^T cell subsets in metabolic related diseases such as type 2 diabetes mellitus and coronary artery disease has long been investigated [18–20]. Yet, the role of CD4^+^T cell subsets in gout process is poorly understood.

CD4^+^T cell subsets consisting of several subpopulations including T helper (Th, pro-inflammatory) and regulatory T cells (Treg, anti-inflammatory), which play critical roles in various autoimmune and inflammatory diseases [21]. Th17/Treg equilibrium is regarded as a crucial component of immunological homeostasis [22]. Imbalance of Th17 and Treg cells in a number of autoimmune disorders has been demonstrated, particularly in systemic lupus erythematosus (SLE) and rheumatoid arthritis (RA) [23, 24]. Some recent studies demonstrated that CD4^+^T cell subsets are also closely associated with gout [12, 25], and indicated a significant factor of CD4^+^T cell subpopulations in gout [25]. Nevertheless, the precise mechanism was not established.

This study analyzed clinical and basic laboratory characteristics of early-onset and late-onset gout patients. Additionally, our study investigated the differences of circulating lymphocyte and CD4^+^T cell subpopulations, and serum cytokines among early-onset gout, late-onset gout and healthy controls (HCs), and was designed to explore the role of CD4^+^T cell subpopulations, particularly Th17 and Treg cells and their corresponding serum cytokines in the pathogenesis of gout.

## Materials and methods

### Participants

We recruited gout inpatients admitted to the Department of Rheumatology in the Second Hospital of Shanxi Medical University (Taiyuan, China) from September 2019 to January 2023. One hundred thirty-three gout patients fulfilled the 2015 American College of Rheumatology/European League Against Rheumatism classification criteria [26], and 6 of them were excluded according to the exclusion criteria: comorbid autoimmune diseases, a history of tumors and severe infections. All gout patients except one were male, so we skipped the 1 female gout patient. In total, this study included 126 gout patients. Additionally, 77 healthy individuals, gender and age matched with gout patients, were from the Physical Examination Centre of our hospital and enrolled as HCs. Ethics approval of this study was granted by the Ethics Committee of the Second Hospital of Shanxi Medical University [Approval (2023) YX No. (156)].

**Fig. 1:**
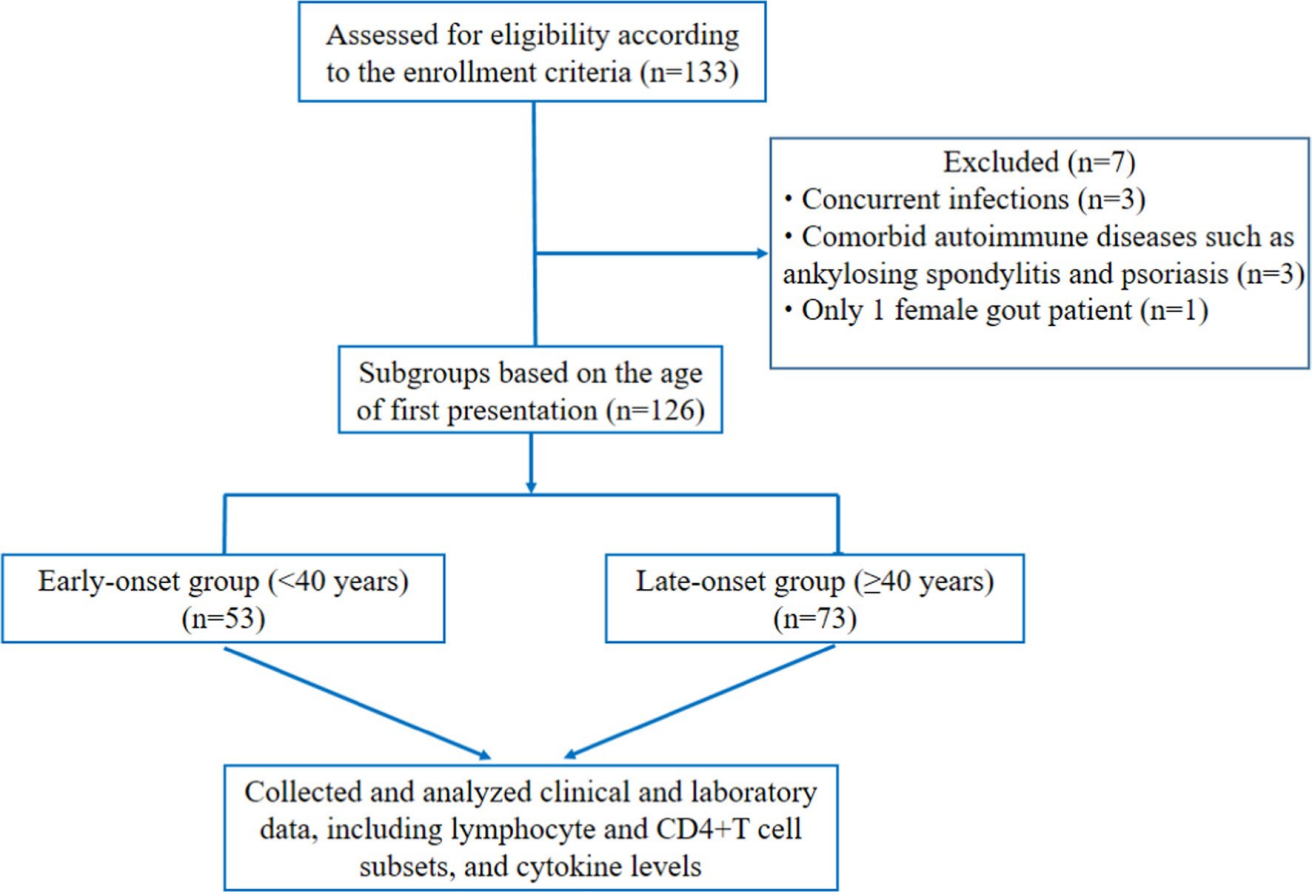
Study flowchart

Based on the age at gout onset, patients were classified into two groups: early-onset group (the age of first presentation < 40 years) [3, 4], and late-onset group (the age of first presentation ≥ 40 years). All enrolled patients were during active gout attack, and untreated with UA lowering therapy or hormonal treatments for this gout flares (Fig. 1).

### Clinical data

The demographics and disease features including gender, age, body mass index (BMI), gout duration time, clinical manifestations and family history of gout were retrospectively collected for all patients. The presence of comorbidities such as CKD and CVD, and the lifestyle were also collected. The laboratory data such as blood cell counts, inflammatory markers, blood lipid, liver and renal function, percentage and absolute counts of lymphocyte and CD4^+^T cell subpopulations, and cytokine levels in the peripheral blood were collected. Fresh blood samples were collected after fasting in the following morning after admission to detect all the above laboratory data.

### Analysis of circulating lymphocyte and CD4^+^T cell subpopulations

The absolute numbers of circulating CD4^+^T/CD8^+^T/B/natural killer (NK) cells were determined by modified one-platform flow cytometry method. Firstly, whole blood fully mixed with anticoagulant was added into two BD Trucount tubes (BD Biosciences, Franklin Lakes, NJ, USA) numbered A and B by reverse pipetting, respectively. Subsequently, anti-CD3/CD4/CD8/CD45 antibodies were added to identify T lymphocyte, while anti-CD3/CD16/CD56/CD45/CD19 antibodies were added to identify B lymphocyte and NK cells.

For analysis of circulating CD4^+^T cell subpopulations (Th1, Th2, Th17 and Treg cells), anticoagulant blood sample was mixed with phorbol myristate acetate, ionomycin and GolgiStop to stimulate Th1/Th2/Th17 cells. Next, the mixture was stained with anti-CD4 antibodies, and mixed with freshly prepared fixation/permeabilization solution. Finally, anti-interferon (IFN)-γ/IL-4/IL-17 antibodies were added to identify Th1/Th2/Th17 cells. Similarly, anticoagulant blood sample was surface-labeled with anti-CD4 and anti-CD25, thoroughly mixed with freshly prepared fixation/permeabilization solution, and followed by intracellular staining with anti-FOXP3 to identify Treg cells.

**Fig. 2:**
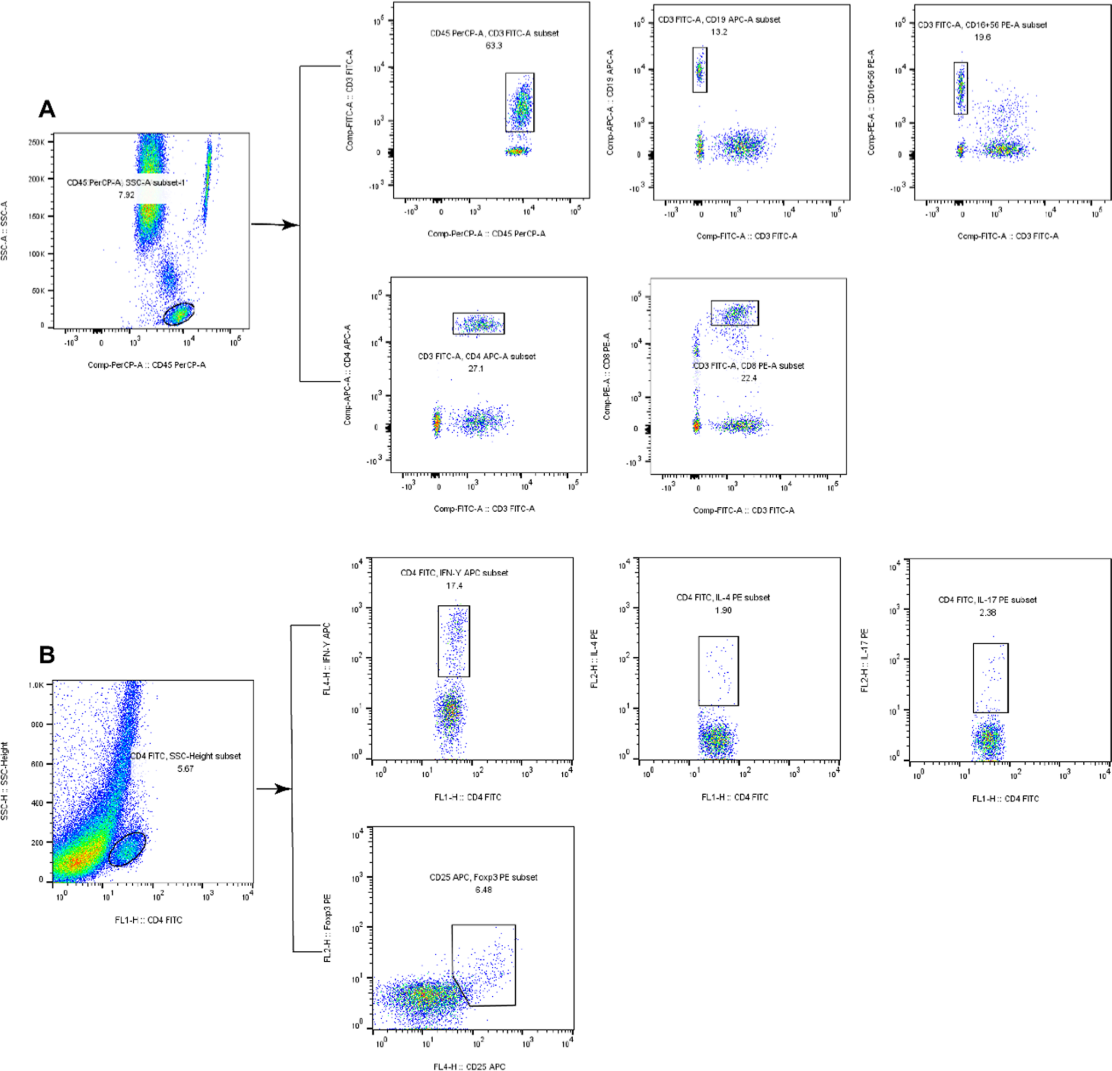
Representative diagram of the gating for flow cytometric analysis of lymphocyte and CD4^+^T subsets. **A** represents flow cytometry analysis of peripheral lymphocytes. T cells: CD45^+^CD3^+^CD19^−^, B cells: CD45^+^CD3^−^CD19^+^, NK cells: CD45^+^CD3^−^CD16^+^CD56^+^, CD4^+^T cells: CD45^+^CD3^+^CD4^+^, CD8^+^T cells: CD45^+^CD3^+^CD8^+^. **B** represents flow cytometry analysis of CD4^+^T cell subsets. Th1 cells: CD4^+^IFN-γ^+^, Th2 cells: CD4^+^IL-4^+^, Th17 cells: CD4^+^IL-17^+^, Treg cells: CD4^+^CD25^+^Foxp3^+^

The immunofluorescence antibodies utilized in this study were procured from BD Biosciences (Franklin Lakes, NJ, USA), and all blood samples were prepared in adherence to the manufacturer's guidelines for mixing, incubation, and washing. Within 24 h of sample collection, detection was carried out using FACSCalibur flow cytometry and BD Multitest software (BD Biosciences, Franklin Lakes, NJ, USA). (Fig. 2).

### Cytokine levels detected by cytometric bead array

The levels of serum cytokines: IFN-γ, tumor necrosis factor (TNF)-α, IL-2, IL-4, IL-6, IL-10 and IL-17 were measured by cell microsphere array using flow cytometry. And cytometric bead array kits (JIANGXI CELLGENE BIOTECH CO., LTD) were applied according to the manufacturer’s recommendations. The results were displayed as pg/ml.

### Statistical analysis

For categorical variables presented as frequencies, Chi-squared test were used for comparison between groups. Continuous numerical variables were displayed as mean ± standard deviation and median (interquartile range). For normally distributed variables, the independent sample *t*-test and analysis of variance method were utilized to compare two groups and multi-groups, respectively. For non-normal distribution variables, the Mann–Whitney *U* test was utilized to compare two groups, while Kruskal–Wallis *H* test was employed to compare multi-groups. The Pearson’s or Spearman’s correlation was employed to ascertain the correlation of two clinical indicators. The receiver operating characteristic (ROC) curve was performed to find optimum cutoff value and the validity of a variable, while the area under the curve (AUC) was utilized to assess the prediction effect. In the study, a *p*-value (two-tailed) < 0.05 was determined to be statistically significant difference. In this study, SPSS software (version 23.0; SPSS Inc., Chicago, IL, USA) and GraphPad Prism software (version 9.0; GraphPad Software Inc., San Diego, CA, USA) were utilized for the statistical analyses.

## Results

### Clinical and demographic features

The demographics feature, lifestyle, disease characteristics and laboratory data of 126 gout patients (53 early-onset gout and 73 late-onset gout) are presented in Table 1. Early-onset gout showed significantly higher BMI, while late-onset gout had a higher frequency of swelling and pain in the first metatarsophalangeal joint, hypertension and CVD, but the two groups did not statistically differ in the frequency of diabetes or CKD.

**Table 1:** Characteristics of patients with early-onset and late-onset gout

	Early-onset gout (*n* = 53)	Late-onset gout (*n* = 73)	*P*-value
Demographic			
Male, *n* (%)	53 (100.0%)	73 (100.0%)	1.000
Age (years)^b^	31.00 (27.00–36.00)	50.00 (46.00–60.00)	< 0.001***
BMI (kg/m^2^)^a^	27.99 ± 3.88	25.72 ± 3.00	< 0.001***
Disease duration (months)^b^	48.00 (24.00–60.00)	36.00 (8.00–96.00)	0.957
Clinical manifestations			
Swelling and pain in the first metatarsophalangeal joint, *n* (%)	23 (43.40%)	59 (67.1%)	0.011*
Tophus, *n* (%)	7 (13.2%)	6 (8.2%)	0.388
Family history of gout, *n* (%)	8 (15.1%)	9 (12.3%)	0.793
Comorbidities			
Hypertension, *n* (%)	10 (18.9%)	28 (38.4%)	0.020*
Diabetes, *n* (%)	1 (1.9%)	8 (11.0%)	0.079
CKD, *n* (%)	2 (3.8%)	8 (11.0%)	0.190
CVD, *n* (%)	0 (0.0%)	9 (12.3%)	0.010*
Lifestyle			
Smoking, *n* (%)	27 (50.9%)	46 (63.0%)	0.203
Drinking, *n* (%)	27 (50.9%)	48 (65.8%)	0.102
Laboratory characteristics			
ESR (mm/h)^b^	30.00 (13.00–72.00)	24.00 (13.00–43.00)	0.300
CRP (mg/ml)^b^	16.00 (3.42–43.50)	11.50 (3.73–25.70)	0.334
D-dimer (ug/L)^b^	155.00 (70.00–377.00)	185.00 (78.00–374.00)	0.707
Fibrinogen (mmol/L)^b^	3.96 (3.06–4.91)	3.66 (3.13–4.52)	0.267
WBC (*10^9/L)^b^	8.53 (6.07–10.32)	7.34 (5.82–8.98)	0.075
LY (*10^9/L)^b^	2.14 (1.74–2.80)	1.85 (1.34–2.27)	0.006**
N (*10^9/L)^b^	5.38 (3.17–7.69)	4.64 (3.46–6.25)	0.500
M (*10^9/L)^b^	0.56 (0.47–0.76)	0.48 (0.33–0.59)	0.001**
Hb (g/L)^b^	149.00 (139.00–153.00)	144.00 (133.00–153.00)	0.299
PLT (*10^9/L)^b^	293.00 (246.00–351.00)	238.00 (193.00–316.00)	0.002**
Liver function			
ALT (U/L)^b^	33.40 (25.70–51.60)	24.40 (17.00–35.60)	0.001**
AST (U/L)^b^	21.60 (16.40–30.90)	20.00 (16.60–27.80)	0.388
ALP (U/L)^b^	88.00 (78.00–106.00)	85.50 (72.00–106.50)	0.171
GGT (U/L)^b^	48.60 (32.00–102.80)	36.15 (26.75–51.00)	0.011*
TBIL (μmol/L)^b^	10.90 (8.60–13.60)	13.55 (9.60–17.250)	0.014*
Renal function			
BUN (mmol/L)^b^	4.30 (3.60–4.90)	5.50 (4.60–6.40)	< 0.001***
Cr (μmol/L)^b^	76.00 (69.00–83.00)	78.00 (68.00–90.00)	0.168
UA (μmol/L)^a^	576.38 ± 132.47	516.54 ± 116.61	0.008**
Serum lipid level			
Total cholesterol (mmol/L)^a^	4.35 ± 0.87	4.25 ± 0.88	0.527
Triglycerides (mmol/L)^b^	1.86 (1.43–2.46)	1.67 (1.21–2.21)	0.255
HDL (mmol/L)^b^	0.85 (0.76–1.09)	0.98 (0.81–1.23)	0.030*
LDL (mmol/L)^b^	2.66 (2.37–3.24)	2.59 (2.09–2.84)	0.063

BMI, body mass index; CKD, chronic kidney disease; CVD, cardiovascular diseases; ESR, erythrocyte sedimentation rate; CRP, C-reactive protein; WBC, white blood cell; LY, lymphocyte; N, neutrophil; M, monocytes; Hb, hemoglobin; PLT, platelet; ALT, alanine transaminase; AST, aspartic transaminase; ALP, alkaline phosphatase; GGT, gamma-glutamyl transpeptidase; TBIL, total bilirubin; BUN, blood urea nitrogen; Cr, creatinine; UA, uric acid; LDL, low density lipoprotein; HDL, high density lipoprotein

*P < 0.05, **P < 0.01, ***P < 0.001

^a^Results are expressed as the mean ± standard deviation

^b^Results are expressed as the median and 25th and 75th percentiles

Early-onset and late-onset gout had no statistically significant difference in laboratory indicators for gout disease activity, including erythrocyte sedimentation rate (ESR), C-reactive protein (CRP), D-dimer and plasma fibrinogen. Notably, early-onset gout had higher levels of serum uric acid (UA) than the late-onset, with an average level of 576.38 ± 132.47 μmol/L. Early-onset gout had significantly lower high-density lipoprotein levels than the late-onset, but the two groups did not show significant differences in other serum lipid levels.

### Correlation analysis of UA levels with clinical and laboratory characteristics in gout

The correlation analysis of serum UA levels with clinical and laboratory characteristics in gout patients (Table 2) showed that UA levels were positively correlated with BMI (*r* = 0.203 *p* = 0.023), total cholesterol (*r* = 0.255, *p* = 0.004), triglycerides (*r*_*s*_ = 0.314, *p* < 0.001) and low-density lipoprotein (*r*_*s*_ = 0.296, *p* = 0.001). Moreover, the absolute counts of Treg cells positively correlated with UA levels (*r*_*s*_ = 0.176, *p* = 0.049). However, UA levels had no significant correlation with ESR, CRP, fibrinogen, D-dimer, and absolute counts of circulating lymphocyte and other CD4^+^T subpopulations in gout patients.

**Table 2:** Correlation analysis of serum UA levels with clinical and laboratory characteristics of gout patients

	*r*/*r*_*s*_	*P*-value
UA vs. BMI	0.203	0.023*
UA vs. Disease duration	0.025	0.780
UA vs. ESR	− 0.067	0.453
UA vs. CRP	− 0.057	0.525
UA vs. fibrinogen	− 0.158	0.077
UA vs. D-dimer	− 0.004	0.966
UA vs. Total cholesterol	0.255	0.004**
UA vs. Triglycerides	0.314	< 0.001***
UA vs. HDL	− 0.133	0.139
UA vs. LDL	0.296	0.001**
UA vs. Total T	0.083	0.353
UA vs. Total B	0.133	0.138
UA vs. NK	− 0.113	0.208
UA vs. CD4^+^T	0.1	0.263
UA vs. CD8^+^T	0.002	0.980
UA vs. Th1	− 0.044	0.627
UA vs. Th2	0.047	0.599
UA vs. Th17	0.026	0.776
UA vs. Treg	0.176	0.049*
UA vs. Th17/ Treg	− 0.142	0.111

Statistics: *r* Pearson correlation test; *r*_*s*_ Spearman correlation test

UA, uric acid; BMI, body mass index; ESR, erythrocyte sedimentation rate; CRP, C-reactive protein; HDL, high density lipoprotein; LDL, low density lipoprotein; T, T lymphocyte; B, B lymphocyte; NK, natural killer cell. Th1, T-helper 1 cells; Th2, T-helper 2 cells; Th17, T-helper 17 cells; Treg, regulatory T cells. (*P < 0.05, **P < 0.01, ***P < 0.001)

### Gout patients had an increased ratio of Th17/Treg by decreased Treg cells and increased Th17 cells

**Fig. 3:**
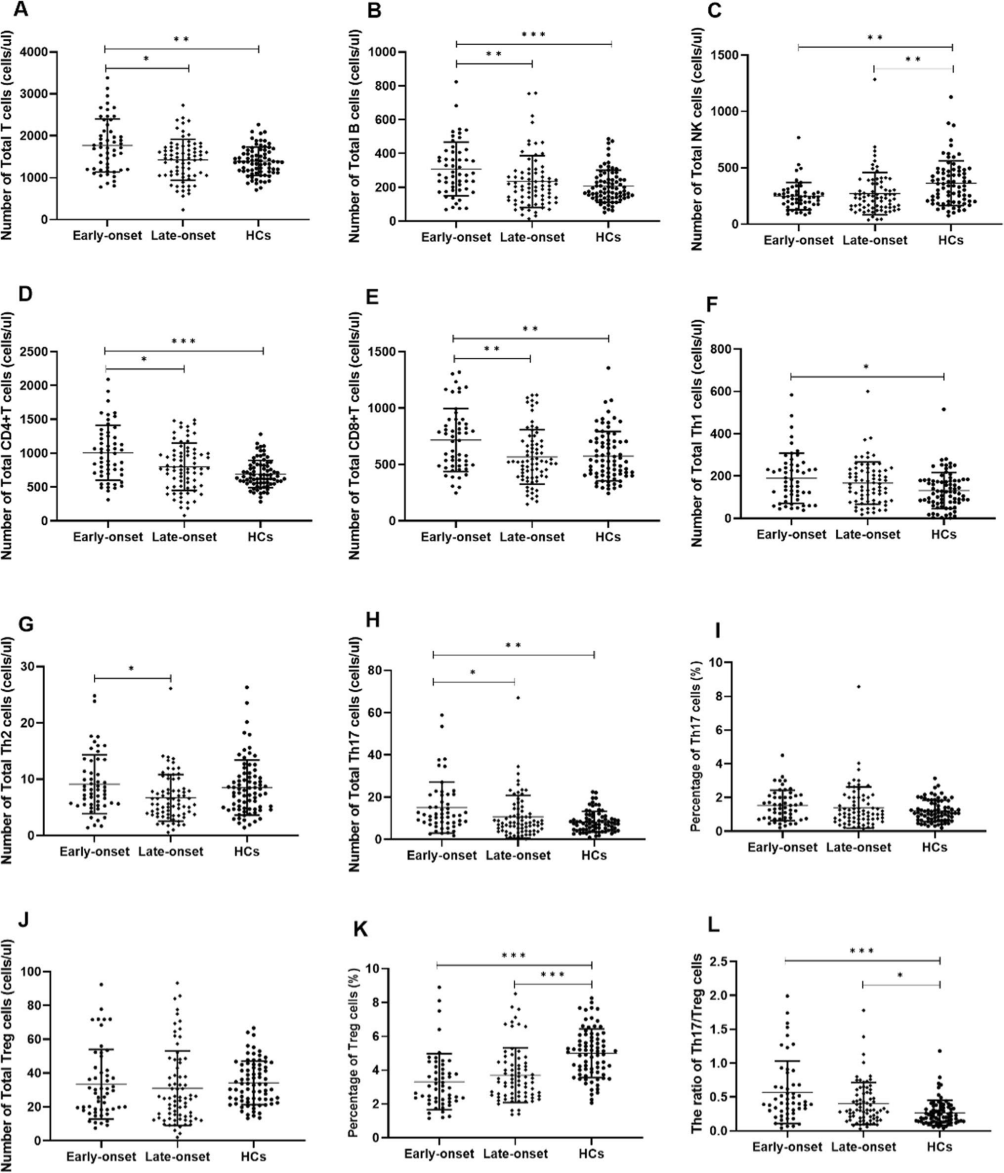
Comparison of absolute numbers of peripheral lymphocyte and CD4^+^T cell subpopulations in early-onset gout, late-onset gout and HCs. **A**–**E** represent the differences in the absolute values of peripheral lymphocyte subsets in three groups (corresponding to Total T, Total B, NK, CD4^+^T, CD8^+^T, respectively). **F**–**L** indicate the differences in absolute values of CD4^+^T cell subsets of three groups (corresponding to Th1, Th2, Th17, Treg cells, Th17/Treg ratio, respectively). HCs, healthy controls. (∗*P* < 0.05, ∗∗*P* < 0.01, and ∗ ∗∗*p* < 0.001)

For circulating lymphocyte subpopulations, early-onset gout differed significantly from HCs with respect to the absolute counts of total T (*p* = 0.002), total B (*p* < 0.001), NK (*p* = 0.002), CD4^+^T (*p* < 0.001) and CD8^+^T cells (*p* = 0.009), while late-onset gout differed significantly from the HCs in the absolute counts of NK cells (*p* = 0.002) (Fig. 3A–E; Supplementary Table S1). These results highly indicated the differences in immune cell disorders in early-onset and late-onset gout.

For the analysis of circulating CD4^+^T cell subpopulations, early-onset gout had significantly elevated absolute counts of Th1 (*p* = 0.011) and Th17 cells (*p* = 0.001) compared to HCs, while the two groups had no significant difference in the absolute counts of Th2 and Treg cells. Notably, late-onset gout and HCs had no statistically significant difference in the absolute counts of Th1, Th2, Th17 and Treg cells. Compared to HCs, the percentage of Treg cells in early- and late-onset gout was significantly lower. Moreover, early-onset gout had significantly higher Th17 cell counts than late-onset gout; the two gout groups had no statistically significant difference in the Treg cells. And Th17/Treg ratio was significantly higher both in early-onset and late-onset gout compared to HCs (*p* < 0.001 and *p* = 0.010) (Fig. 3F–L; Supplementary Table S1). These results suggested that early-onset and late-onset gout differed in imbalanced Th17/Treg homeostasis.

Correlation analysis (Supplementary Figure S1) suggested that Th17 cells had significantly negative correlations with PLT (*r*_*s*_ = 0.186 *p* = 0.036). Treg cells had significantly negative correlations with inflammatory indicators (including ESR, CRP and fibrinogen) and neutrophils. Percentage of Treg cells was negatively related to BMI (*r*_*s*_ = − 0.201 *p* = 0.023). Thus, restoring Th17/Treg balance may be essential to alleviating gout inflammation.

### Gout patients had significantly higher cytokine levels than HCs, and the IL-2 levels in gout patients were positively correlated with Treg cells

Cytokine levels, including IFN-γ, TNF-α, IL-2, IL-4, IL-6, IL-10 and IL-17, were detected in early-onset gout, late-onset gout and HCs. Compared to HCs, early-onset and late-onset gout patients had significantly higher levels of these cytokines (Supplementary Table S2). Nevertheless, early-onset and late-onset gout patients had no significant difference in these cytokine levels.

**Fig. 4:**
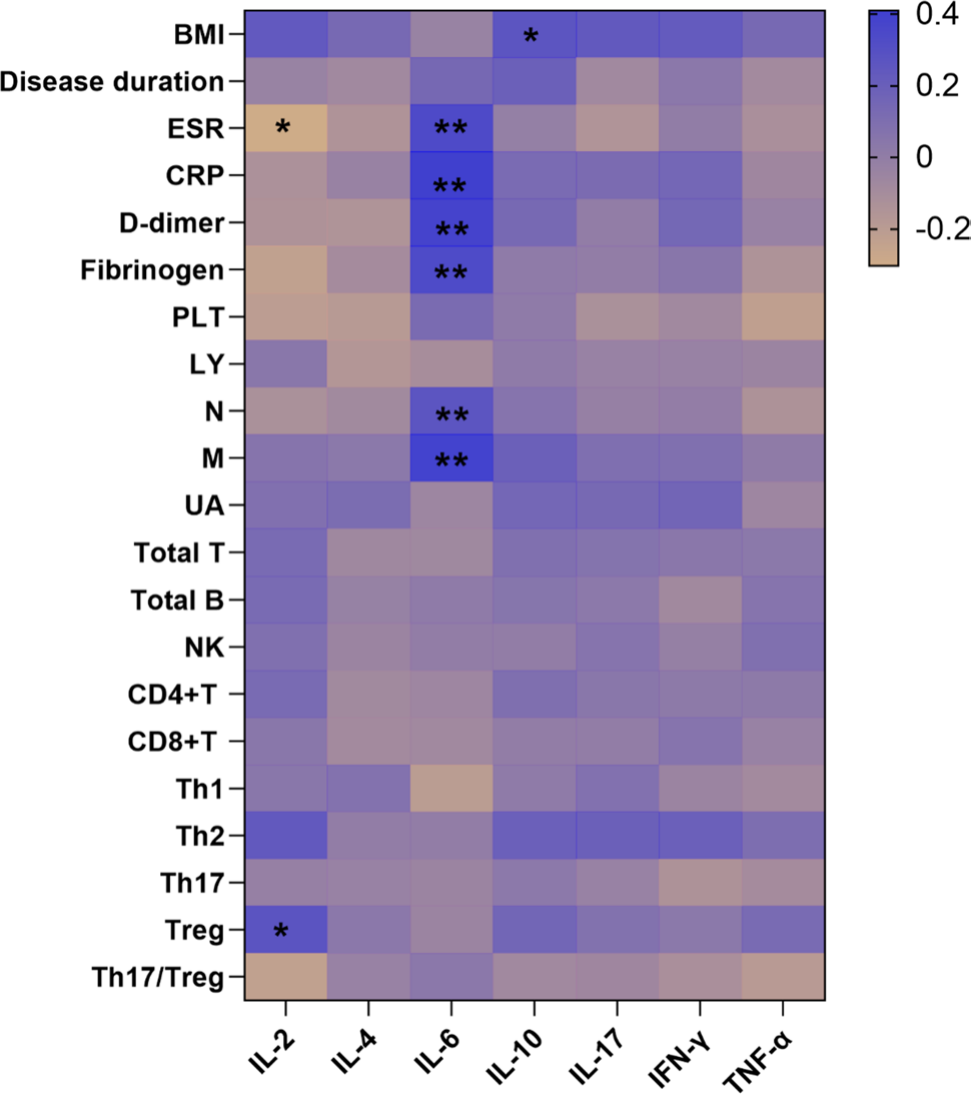
Heatmap of correlation of serum cytokine levels with clinical and laboratory characteristics of gout patients. The IL-6 was positively correlated with many laboratory indicators including ESR, CRP, D-dimer, fibrinogen and monocytes, while IL-10 was positively correlated with BMI. And IL-2 was negatively correlated with ESR, as well as positively correlated with Treg cells. (∗*P* < 0.05, ∗∗*P* < 0.01, and ∗∗∗*P* < 0.001)

The correlation analysis in gout patients (Fig. 4**)** showed that there were significantly negative correlations in the levels of IL-2 and ESR (r_s_ = − 0.303, *p* = 0.014), while IL-10 was positively related to BMI (*r*_*s*_ = 0.261, *p* = 0.035). Notably, IL-6 was positively related to ESR (*r*_*s*_ = 0.332, *p* = 0.007), CRP (*r*_*s*_ = 0.409, *p* = 0.001), D-dimer (*r*_*s*_ = 0.382, *p* = 0.002), fibrinogen (*r*_*s*_ = 0.345, *p* = 0.005), neutrophils (*r*_*s*_ = 0.266 *p* = 0.032) and monocytes (*r*_*s*_ = 0.390 *p* = 0.001). These findings suggested that the pathophysiology of gout may be mediated by various cytokines.

The correlation analysis of serum cytokine levels with circulating lymphocyte and CD4^+^T cell subpopulations in gout patients (Fig. 4) described that IL-2 levels were positively related to the absolute counts of Treg cells (*r*_*s*_ = 0.283, *p* = 0.022). Other serum cytokines did not correlate significantly with circulating lymphocyte and CD4^+^T cell subpopulations.

### Gout patients without tophus had an increased ratio of Th17/Treg by increased Th17 cells, which had no significant correlation with IL-2

**Fig. 5:**
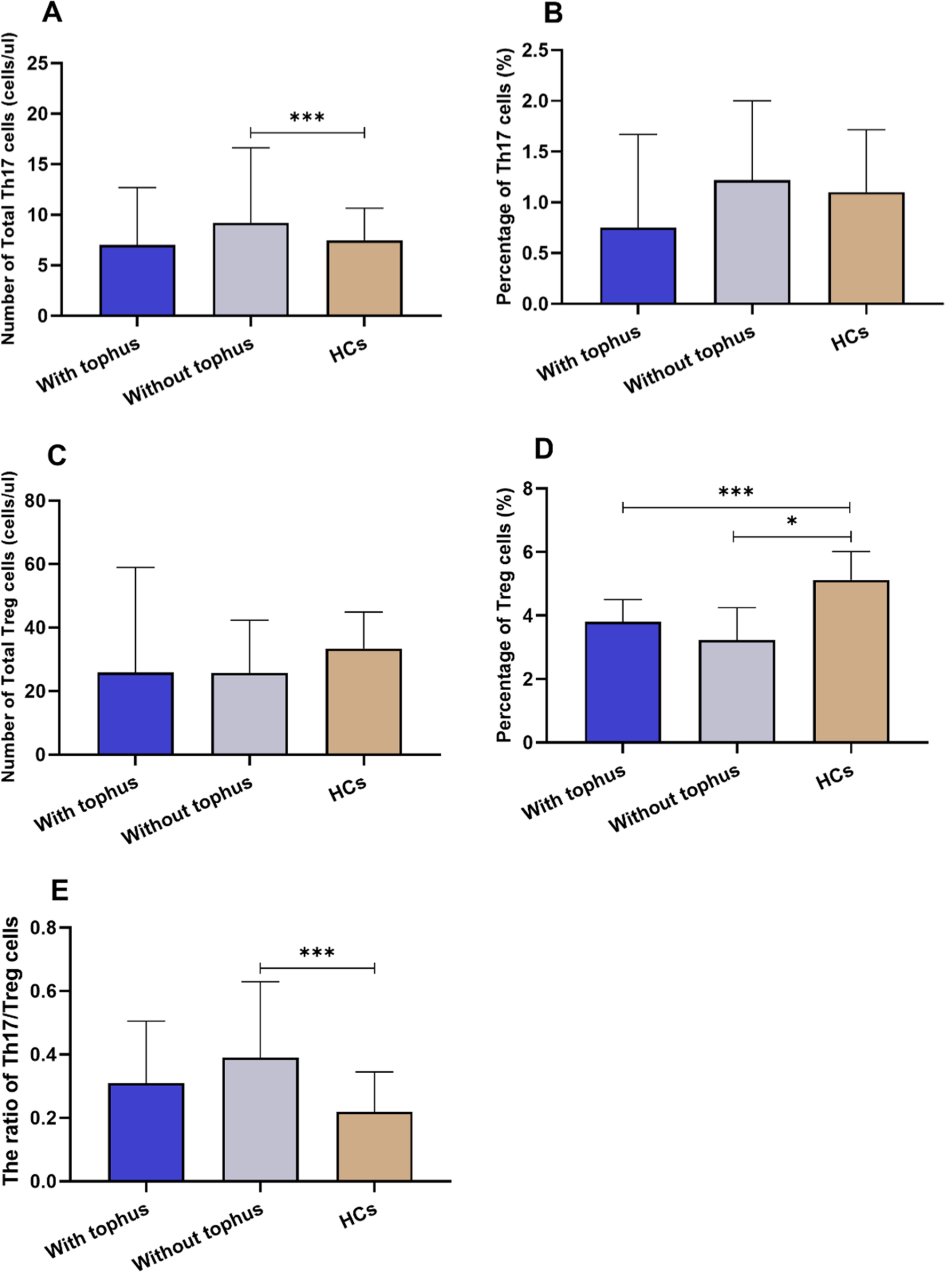
Comparison of absolute numbers of peripheral Th17 and Treg cells in gout without tophus, gout with tophus and HCs. Abbreviations: HCs, healthy controls. (∗*P* < 0.05, ∗∗*P* < 0.01, and ∗∗∗*p* < 0.001)

We further subdivide 126 patients into gout with tophus and gout without tophus, then analyzed circulating Th17 and Treg cells among gout with tophus, gout without tophus and HCs. We found that gout without tophus had significantly higher number of Th17 cells (*p* < 0.001) and Th17/Treg ratio (*p* < 0.001) than HCs; gout with tophus and gout without tophus both exhibited lower percentage of Treg cells than HCs. Nevertheless, the levels of Th17 and Treg cells had no significant difference in gout with and without tophus (Fig. 5).

Then, we compared cytokine levels, including IFN-γ, TNF-α, IL-2, IL-4, IL-6, IL-10 and IL-17 among gout with tophus, gout without tophus and HCs. Compared to HCs, gout patients with and without tophus had significantly higher levels of these cytokines (Supplementary Table S3). Yet, gout patients with and without tophus had no significant difference in these cytokine levels. The correlation analysis of serum cytokine levels with circulating Th17 and Treg cells in gout with tophus and gout without tophus showed that these cytokine levels had no significant correlation with Th17 and Treg cells (Supplementary Table S4 and S5).

### ROC curve analysis for predicting the presence of tophus in gout.

**Fig. 6:**
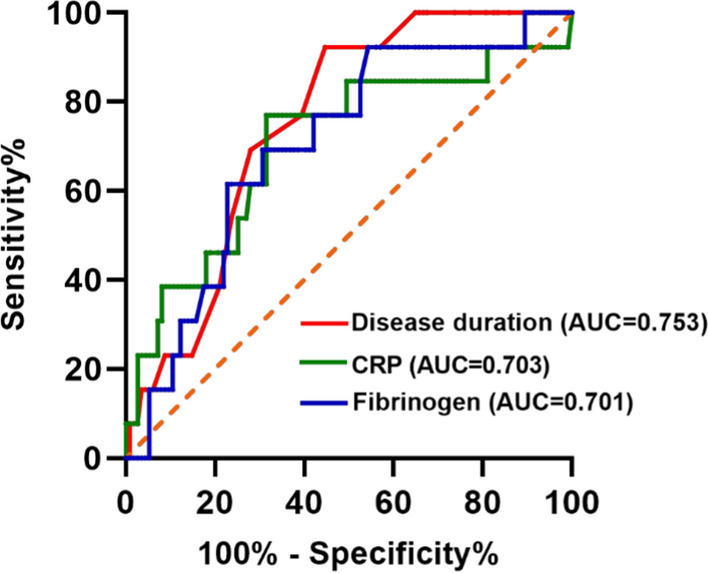
Receiver operating characteristic (ROC) curve of indicators for the presence of tophus in gout. (Red) the area under the ROC curve (AUC) of disease duration was 0.753, and its sensitivity and specificity were 0.923 and 0.558, respectively. (Green) the AUC of CRP was 0.703, and its sensitivity and specificity were 0.769 and 0.681, respectively. (Blue) the AUC of fibrinogen was 0.701, and its sensitivity and specificity were 0.615 and 0.770, respectively (color figure online)

ROC analysis was utilized to assess the ability of indicators to predict the presence of tophus in gout. The results showed that three of them, including disease duration, CRP and fibrinogen, had moderate predictive performances for tophus in gout (Supplementary Table S6). The AUCs for disease duration, CRP and fibrinogen in distinguishing tophus in gout were 0.753 (sensitivity of 0.923, specificity of 0.558, *p* = 0.003), 0.703 (sensitivity of 0.769, specificity of 0.681, *p* = 0.017) and 0.701 (sensitivity of 0.615, specificity of 0.770, *p* = 0.018) (Fig. 6). The optimal cut-off values of disease duration, CRP and fibrinogen in distinguishing the presence of tophus in gout patients were 42 months, 20.65 mg/ml and 4.625 mmol/L, respectively.

## Discussion

The translational study in immunity disorder found that there were differences in Th17/Treg imbalance between patients with early-onset and late-onset gout. Early-onset gout had significantly elevated Th17 cells than late-onset gout and HCs; the percentage of Treg cells in early-onset and late-onset gout was significantly decreased than HCs. And increased serum cytokine levels, especially IL-2, may play an essential role in that. In addition, gout without tophus had significantly higher number of Th17 cells and Th17/Treg ratio than HCs, which revealed the fact that gout had a higher inflammatory response in the early stages of an attack. Moreover, disease duration, CRP and fibrinogen can predict the presence of tophus in gout.

In our study, correlation analysis showed that UA concentration was positively related to BMI and serum lipid profiles, including total cholesterol, low-density lipoprotein and triglycerides. Nevertheless, the lymphocyte counts and cytokine levels were unrelated to UA levels. Thus, lifestyles such as high fructose intake and high genetic risk may play more prominent roles than immune cells in elevated serum UA levels [27]. In addition, we found that early-onset gout patients had significantly higher UA levels and BMI than the late-onset, but the two groups did not statistically differ in the frequency of diabetes or CKD. In general, renal function declines with age, yet the onset age of type 2 diabetes and CKD shows a younger trend [28, 29]. Thus, young people with hyperuricemia and gout should pay more attention to the control of weight, blood glucose levels and protection of renal function.

This study suggests that early-onset and late-onset gout differ in Th17/Treg imbalance. In other words, an increased ratio of Th17/Treg in early-onset gout may be due to an increase in Th17 cells, as well as in late-onset gout could mainly result from a decrease in Treg cells. In recent years, Th17 and Treg cells have gained attention in the immunopathological research of numerous diseases. Imbalance of Th17/Treg has been reported to be associated with some inflammatory and autoimmune diseases, such as RA and SLE [23, 24, 30]. Similarly, Luo et al. [31] and Zhao et al. [25] both reported that acute gout patients had elevated counts of circulating Th17 cells and Th17/Treg ratio. Furthermore, experimental studies demonstrated that rats with acute gout flare induced by MSU crystals had elevated levels of inflammatory Th17 cells, and the elevated Th17/Treg ratio was consistent with the inflammation development of gouty arthritis, suggesting that Th17/Treg imbalance is involved in the pathogenesis of gout attack [32]. Activated Th17 cells may secrete IL-17 which can cause neutrophils promptly accumulated to accelerate the release of inflammatory cytokines, and exacerbate the joint inflammation [25]. Treg cells have been demonstrated to lessen gout-related immune response and bone damage by secreting cytokines immunosuppressive such as IL-10 and transforming growth factor (TGF)-β [6]. Therefore, controlling the balance of Th17/Treg cells is essential for preventing the emergence of inflammatory diseases [33].

Changes in Th17 and Treg cells, along with elevated levels of circulating inflammatory cytokines in gout patients, indicate that gout effects the entire body as a systemic disease rather than just being a localized form of arthritis [32]. In gout, MSU crystals stimulate the macrophage by activating NLRP3 inflammasome, triggering the release of pro-inflammatory cytokines [10, 34]. Although IL-1β plays a crucial role in the pathogenesis of the pathogenesis of gout flares, other cytokines such as IL-6, TNF-α, and TGF-β are also involved in that [16, 31, 35]. In the present study, the IL-6 levels were elevated in gout patients, and positively related to ESR, CRP, D-dimer, fibrinogen, neutrophils and monocytes. IL-6, as a pro-inflammatory cytokine, amplifies the inflammatory process and possibly leads to bone damage [36]. For Th17/Treg balance, IL-6 together with TGF-β induce naïve T cells to differentiate into Th17 cells; conversely, IL-6 inhibits the TGF-β-induced FOXP3 expression and prevents Treg differentiation [37, 38]. Cavalcanti et al. [36] found that IL-6 levels were related to increased ESR and CRP in gout patients, and had a significant association with the presence of deformities and tophus. Therefore, therapeutic regimens targeting IL-6 inhibition attenuate the inflammatory response, thereby restoring Th17/Treg balance, which may be critical in the treatment of early-onset gout.

CD4^+^CD25^+^FOXP3^+^Tregs are significant to the induction and maintenance of immune homeostasis and tolerance [39]. Cytokines (especially TGF-β and IL-2) are required in the development and differentiation of both induced and thymic Tregs [39, 40]. IL-2 was the first cytokine molecularly cloned in 1983 [41], and is a pleiotropic cytokine, which orchestrates immune responses via the promotion of differential growth and activation of Tregs and effector T cells to maintain immunological homeostasis [42]. Some studies have reported that IL-2 is a crucial cytokine which keeps Treg cells from differentiating, proliferating, and functioning [42, 43]. TCR-stimulated Treg cells were unable to produce IL-2, but IL-2 provided by activated effector T cells was necessary for their survival and proliferation [44]. In our study, the percentage of Treg cells in early-onset and late-onset gout was significantly lower than HCs. And Treg cells had significantly negative correlations with inflammatory indicators and neutrophils. The elevated IL-2 levels in gout patients were negatively correlated with ESR levels. Furthermore, IL-2 levels positively correlated with Treg cells IL-2 levels, suggesting restoring Treg cells levels may be a potential treatment for gout. Currently, low-dose IL-2 therapy tried to treat various autoimmune diseases including RA, primary Sjögren's syndrome and SLE, has been shown to increase the number of CD4^+^CD25^+^FOXP3^+^Treg cells and achieved therapeutic results [24, 42, 45]. Similarly, low-dose IL-2 therapy based on stimulating Treg cells proliferation and restore immune tolerance may offer new directions for exploring the treatment of late-onset gout.

The tophus demonstrates a multifaceted, well-organized chronic inflammatory tissue response to MSU monohydrate crystals, which involves both innate and adaptive immune cells [46]. And the formation of tophus can cause joint deformities, joint injury, fracture, and skin rupture or infection [47]. In our study, gout without tophus had significantly higher number of Th17 cells and Th17/Treg ratio than HCs, while gout with tophus and gout without tophus both exhibited lower percentage of Treg cells than HCs. Similarly, Luo et al. [31] found that acute gout had significantly higher absolute number and percentage of Th17 and Th22 cells than intercritical gout and HCs. These findings implied that gout without tophus had a more pronounced inflammatory response. The ROC curves showed that disease duration, CRP and fibrinogen had moderate predictive performances for tophus in gout, which indicated that tophus was chronic inflammatory tissue response and mostly the result of chronic recurrent gout attacks [48]. Further studies with the aid of single-cell RNA sequencing and other advanced new technologies may accurately explore the Th17/Treg imbalance in different disease states of gout [35].

This study has some limitations. Firstly, the included patients were from the same medical center and all were during active gout attack. Secondly, these peripheral blood samples were obtained without doing in vitro experiments to verify our findings. Thirdly, we were unable to quantify CD4^+^T cell subpopulations and cytokines in synovial fluid or tophus, which would more accurately reflect their production in acute gout. Lastly, it is necessary to corroborate the direct evidence in suitable animal models for the contribution of imbalanced Th17/Treg in the pathogenesis of gout.

## Conclusion

In summary, this study suggests that early-onset and late-onset gout differ in Th17/Treg imbalance. And higher level of serum cytokines, especially IL-2, may play an essential role in that. Restoring Th17/Treg balance may be a crucial way to improve the prognosis of gout patients. In the future, in-depth studies should be conducted to determine whether differences in Th17/Treg imbalance and IL-2 in the different age of onset- gout are caused by T-cell senescence and disruption of immune tolerance with aging.

## Supplementary Information

Below is the link to the electronic supplementary material.

Supplementary file1 (DOCX 176 KB)

## Data Availability

Raw data used during the current study are available from the corresponding author, upon reasonable request.

## Abbreviations

MSU: Monosodium urate
CVD: Cardiovascular disease
CKD: Chronic kidney disease
NETs: Neutrophil extracellular traps
Th: T helper
Treg: Regulatory T
SLE: Systemic lupus erythematosus
RA: Rheumatoid arthritis
HCs: Healthy controls
BMI: Body mass index
NK: Natural killer
IFN: Interferon
TNF: Tumor necrosis factor
IL: Interleukin
ROC: Receiver operating characteristic
AUC: Area under the curve
ESR: Erythrocyte sedimentation rates
CRP: C-reactive protein
UA: Serum uric acid
TGF: Transforming growth factor
