# Supplementary material for: Elevated serum IL-2 and Th17/Treg imbalance are associated with gout
Source: Clin Exp Med. 2024 Jan 19;24(1):9. doi: 10.1007/s10238-023-01253-4 (PMC10799120; doi:10.1007/s10238-023-01253-4)
Supplement: Supplementary file 1 — Supplementary file1 (DOCX 176 KB) [file 10238_2023_1253_MOESM1_ESM.docx]

**Elevated serum IL-2 and Th17/Treg imbalance are associated with gout**

Xiaoyu Zi^1,2^, Ronghui Su^1,2^, Rui Su^1,2^, Hui Wang^1,2^, Baochen Li^1,2^, Chong Gao^3^, Xiaofeng Li^1,2^ and Caihong Wang^1,2*^

^1^ Department of Rheumatology, the Second Hospital of Shanxi Medical University, Taiyuan, Shanxi, China;

^2^ Shanxi Key Laboratory of Immunomicroecology, Taiyuan, Shanxi, China;

^3^ Pathology, Joint Program in Transfusion Medicine, Brigham and Women’s Hospital/Children’s Hospital, Harvard Medical School, Boston, MA, United States

Correspondence: Caihong Wang, the Second Hospital of Shanxi Medical University, No. 382 Wuyi Road, Xinghualing District, 030000 Taiyuan, Shanxi, China. snwch@sina.com.

Journal name: Clinical and Experimental Medicine

**Supplementary Table S1** Peripheral lymphocyte and CD4^+^T cell subpopulations in early-onset gout, late-onset gout and HCs.

|  | Early-onset gout (A)（n=53） | Late-onset gout (B)（n=73） | HCs (C)（n=77） | ***P***-value | A vs. B | A vs. C | B vs. C |
| --- | --- | --- | --- | --- | --- | --- | --- |
| Total T (cells/μL)^a,b^ | 1769.13±633.90 | 1436.68 ±482.99 | 1376.76(1144.22-1630.77) | 0.002** | 0.015* | 0.002** | 1.000 |
| T%^a,b^ | 73.71±7.03 | 72.59±8.57 | 70.38(64.00-75.25) | 0.017* | 0.774 | 0.016* | 0.219 |
| Total B (cells/μL)^b^ | 276.13(191.02-406.08) | 207.64(139.00-287.20) | 177.89(142.00-246.00) | <0.001*** | 0.007** | <0.001*** | 1.000 |
| B%^b^ | 13.51(9.37-16.19) | 10.98(8.15-14.18) | 10.31(7.00-13.16) | 0.018* | 0.349 | 0.014* | 0.522 |
| NK (cells/μL)^b^ | 239.78(181.04-282.52) | 234.98(151.56-358.96) | 340.59(204.94-469.00) | <0.001*** | 1.000 | 0.002** | 0.002** |
| NK%^b^ | 9.24(7.23-14.39) | 12.57(8.77-17.18) | 17.00(12.65-21.40) | <0.001*** | 0.069 | <0.001*** | 0.002** |
| CD4^+^T cells^a,b^ | 1007.61±404.37 | 806.64 ±345.85 | 654.61(561.32-821.68) | <0.001*** | 0.029* | <0.001*** | 0.078 |
| CD4^+^T% ^a^ | 40.95±7.33 | 39.36±10.40 | 35.37 ± 7.90 | 0.001** | 0.317 | <0.001*** | 0.006** |
| CD8^+^T cells^b^ | 713.59(488.62-864.86) | 524.77(396.38-707.71) | 529.29(411.00-705.17) | 0.004** | 0.008** | 0.009** | 1.000 |
| CD8^+^T%^b^ | 28.64(26.00-33.33) | 27.94(21.33-37.9) | 28.27(22.69-34.32) | 0.606 | - | - | - |
| CD4^+^T/CD8^+^T cells^b^ | 1.42(1.06-1.77) | 1.50(0.89-2.09) | 1.31(0.90-1.67) | 0.198 | - | - | - |
| Th1 (cells/μL)^b^ | 172.26(103.21-249.57) | 163.96(84.78-224.90) | 125.72(77.17-178.16) | 0.007** | 1.000 | 0.011* | 0.055 |
| Th1%^b^ | 19.88(12.64-27.00) | 21.40(14.54-30.21) | 16.79(12.96-26.62) | 0.155 | - | - | - |
| Th2 (cells/μL)^b^ | 8.11(5.50-11.39) | 5.63(3.84-8.14) | 7.60(4.72-10.50) | 0.009** | 0.014* | 1.000 | 0.052 |
| Th2%^b^ | 1.00(0.62-1.07) | 0.78(0.62-1.04) | 1.11(0.80-1.60) | <0.001*** | 1.000 | 0.004** | <0.001*** |
| Th17 (cells/μL)^b^ | 11.39(7.76-20.92) | 7.95(4.15-13.35) | 7.47(4.56-10.65) | 0.001** | 0.013* | 0.001** | 1.000 |
| Th17%^b^ | 1.40(0.78-2.10) | 1.07(0.70-2.00) | 1.10(0.68-1.66) | 0.156 | - | - | - |
| Treg (cells/μL)^b^ | 28.10(18.41-41.60) | 25.10(14.34-42.94) | 33.37(24.40-44.84) | 0.053 | - | - | - |
| Treg %^b^ | 2.95(2.24-4.05) | 3.30(2.50-4.42) | 5.12(3.88-5.94) | <0.001*** | 0.451 | <0.001*** | <0.001*** |
| Th17/Treg^b^ | 0.41(0.29-0.68) | 0.31(0.19-0.56) | 0.22(0.14-0.34) | <0.001*** | 0.127 | <0.001*** | 0.010* |

Abbreviations：HCs, healthy controls; T, T lymphocyte; B, B lymphocyte; NK, natural killer cell; Th1, T-helper 1 cells; Th2, T-helper 2 cells; Th17, T-helper 17 cells; Treg, regulatory T cells. (* ***P*** < 0.05, ** ***P*** < 0.01, *** ***P*** < 0.001)

**^a^Results are expressed as the mean ± standard deviation.**

**^b^Results are expressed as the median and 25th and 75th percentiles.**

**Supplementary Table S2** Serum cytokine levels in in early-onset gout, late-onset gout and HCs.

|  | Early-onset gout (A) | Late-onset gout (B) | HCs (C) | P-value | A vs. B | A vs. C | B vs. C |
| --- | --- | --- | --- | --- | --- | --- | --- |
| IL-2 (pg/ml) ^b^ | 2.64(1.32-4.01) | 2.98(1.84-3.48) | 1.68(1.58-1.84) | 0.001** | 1.000 | <0.001*** | <0.001*** |
| IL-4 (pg/ml) ^b^ | 2.69(1.85-5.51) | 2.46(1.12-4.19) | 1.32(1.22-1.49) | <0.001*** | 0.289 | <0.001*** | <0.001*** |
| IL-6 (pg/ml) ^b^ | 14.40(8.22-50.59) | 17.64(4.94-30.83) | 2.19(1.99-2.9) | <0.001*** | 0.520 | <0.001*** | <0.001*** |
| IL-10 (pg/ml) ^b^ | 6.59(5.35-9.08) | 5.53(3.90-10.53) | 1.76(1.55-1.99) | <0.001*** | 1.000 | <0.001*** | <0.001*** |
| IL-17 (pg/ml) ^b^ | 8.27(3.37-25.06) | 4.54(1.14-22.28) | 0。00(0.00-0.27) | <0.001*** | 0.642 | <0.001*** | <0.001*** |
| INF-γ (pg/ml) ^b^ | 5.95(3.76-9.70) | 3.65(2.48-7.8) | 1.38(1.19-1.63) | <0.001*** | 0．027* | <0.001*** | <0.001*** |
| TNF-α(pg/ml) ^b^ | 3.20(1.41-6.01) | 2.06(1.35-4.56) | 1.17(1.06-1.44) | <0.001*** | 0.757 | <0.001*** | 0.003** |

Abbreviations：HCs, Healthy controls; IL-2, interleukin-2; IL-4, interleukin-4; IL-6, interleukin-6; IL-10, interleukin-10; IL-17, interleukin-17; INF-γ, interferon-γ; TNF-α, tumor necrosis factor-α. (* P <0.05, ** P <0.01, *** P <0.001)

**^b^Results are expressed as the median and 25th and 75th percentiles.**

**Supplementary Table S3** Serum cytokine levels in gout with tophus, gout without tophus and HCs.

|  | Gout with tophus (A) （n=13） | Gout without tophus (B) （n=113） | HCs (C) （n=77） | ***P***-value | A vs. B | A vs. C | B vs. C |
| --- | --- | --- | --- | --- | --- | --- | --- |
| IL-2 (pg/ml) ^b^ | 3.51(2.31-3.51) | 2.64(1.70-3.48) | 1.68(1.58-1.84) | <0.001*** | 0.809 | 0.002** | <0.001*** |
| IL-4 (pg/ml) ^b^ | 2.01(1.43-2.52) | 2.87(1.12-4.90) | 1.32(1.22-1.49) | <0.001*** | 1.000 | 0.005** | <0.001*** |
| IL-6 (pg/ml) ^b^ | 39.06(26.65-39.06) | 14.09(5.64-30.83) | 2.19(1.99-2.9) | <0.001*** | 0.275 | <0.001*** | <0.001*** |
| IL-10 (pg/ml) ^b^ | 5.95(5.43-6.25) | 6.16(3.90-9.43) | 1.76(1.55-1.99) | <0.001*** | 1.000 | <0.001*** | <0.001*** |
| IL-17 (pg/ml) ^b^ | 5.54(5.54-20.68) | 5.08(1.57-25.62) | 0.00(0.00-0.27) | <0.001*** | 1.000 | <0.001*** | <0.001*** |
| INF-γ (pg/ml) ^b^ | 3.83(3.02-9.33) | 5.49(3.19-8.76) | 1.38(1.19-1.63) | <0.001*** | 1.000 | <0.001*** | <0.001*** |
| TNF-α(pg/ml) ^b^ | 2.06(1.30-6.22) | 2.61(1.41-4.63) | 1.17(1.06-1.44) | <0.001*** | 0.940 | 0.001** | <0.001*** |

Abbreviations：HCs, Healthy controls; IL-2, interleukin-2; IL-4, interleukin-4; IL-6, interleukin-6; IL-10, interleukin-10; IL-17, interleukin-17; INF-γ, interferon-γ; TNF-α, tumor necrosis factor-α. (* P <0.05, ** P <0.01, *** P <0.001)

**^b^Results are expressed as the median and 25th and 75th percentiles.**

**Supplementary Table S4** Correlation analysis of Th17, Treg cells and cytokines in gout with tophus.

|  | Th17 (cells/ml) | | Th17% | | Treg (cells/ml) | | Treg% | | Th17/Treg | |
| --- | --- | --- | --- | --- | --- | --- | --- | --- | --- | --- |
|  | r | p | r | p | r | p | r | p | r | p |
| IL-2 (pg/ml) | -0.226 | 0.459 | -0.374 | 0.208 | 0.305 | 0.310 | 0.094 | 0.760 | -0.299 | 0.321 |
| IL-4 (pg/ml) | 0.245 | 0.420 | 0.265 | 0.382 | -0.093 | 0.763 | -0.096 | 0.756 | 0.376 | 0.206 |
| IL-6 (pg/ml) | 0.129 | 0.675 | 0.081 | 0.792 | -0.034 | 0.913 | -0.345 | 0.249 | 0.395 | 0.182 |
| IL-10 (pg/ml) | -0.140 | 0.648 | -0.379 | 0.202 | 0.368 | 0.217 | 0.295 | 0.329 | -0.540 | 0.057 |
| IL-17 (pg/ml) | -0.328 | 0.273 | -0.357 | 0.231 | -0.106 | 0.731 | -0.100 | 0.745 | -0.119 | 0.698 |
| IFN-γ(pg/ml) | -0.053 | 0.863 | -0.106 | 0.730 | 0.028 | 0.928 | 0.184 | 0.547 | -0.060 | 0.844 |
| TNF-α(pg/ml) | -0.076 | 0.806 | 0.003 | 0.993 | 0.272 | 0.368 | 0.429 | 0.143 | -0.076 | 0.804 |

Statistics: Spearman correlation test.

Abbreviations：Th17, T helper 17 cells; Treg, regulatory T cells; IL-2, interleukin-2; IL-4, interleukin-4; IL-6, interleukin-6; IL-10, interleukin-10; IL-17, interleukin-17; INF-γ, interferon-γ; TNF-α, tumor necrosis factor-α.*P<0.05, **P<0.01, ***P<0.001.

**Supplementary Table S5** Correlation analysis of Th17, Treg cells and cytokines in gout without tophus.

|  | Th17 (cells/ml) | | Th17% | | Treg (cells/ml) | | Treg% | | Th17/Treg | |
| --- | --- | --- | --- | --- | --- | --- | --- | --- | --- | --- |
|  | r | p | r | p | r | p | r | p | r | p |
| IL-2 (pg/ml) | -0.078 | 0.411 | -0.103 | 0.277 | 0.033 | 0.726 | 0.043 | 0.651 | -0.150 | 0.113 |
| IL-4 (pg/ml) | -0.051 | 0.594 | -0.112 | 0.239 | 0.050 | 0.602 | 0.012 | 0.897 | -0.135 | 0.153 |
| IL-6 (pg/ml) | -0.046 | 0.628 | -0.030 | 0.753 | -0.149 | 0.114 | -0.158 | 0.095 | 0.054 | 0.573 |
| IL-10 (pg/ml) | -0.037 | 0.693 | -0.017 | 0.861 | -0.110 | 0.246 | -0.113 | 0.235 | 0.044 | 0.640 |
| IL-17 (pg/ml) | -0.045 | 0.639 | -0.063 | 0.509 | -0.003 | 0.976 | -0.060 | 0.529 | -0.032 | 0.735 |
| IFN-γ(pg/ml) | -0.029 | 0.763 | -0.072 | 0.450 | -0.023 | 0.805 | -0.139 | 0.141 | -0.011 | 0.904 |
| TNF-α(pg/ml) | 0.044 | 0.642 | 0.061 | 0.521 | 0.019 | 0.844 | 0.037 | 0.694 | 0.022 | 0.818 |

Statistics: Spearman correlation test.

Abbreviations：Th17, T helper 17 cells; Treg, regulatory T cells; IL-2, interleukin-2; IL-4, interleukin-4; IL-6, interleukin-6; IL-10, interleukin-10; IL-17, interleukin-17; INF-γ, interferon-γ; TNF-α, tumor necrosis factor-α.*P<0.05, **P<0.01, ***P<0.001.

**Supplementary Table S6** The predictive capacity of indicators for the presence of tophus in gout.

|  | AUC | 95% CI | ***P***-value |
| --- | --- | --- | --- |
| Age (years) | 0.418 | 0.223-0.614 | 0.336 |
| BMI (kg/m^2^) | 0.414 | 0.199-0.630 | 0.312 |
| Disease duration (months) | 0.753 | 0.647-0.858 | 0.003** |
| ESR (mm/h) | 0.69 | 0.567-0.812 | 0.026* |
| CRP (mg/ml) | 0.703 | 0.539-0.866 | 0.017* |
| D-dimer (ug/L) | 0.679 | 0.551-0.808 | 0.035* |
| Fibrinogen (mmol/L) | 0.701 | 0.563-0.839 | 0.018* |
| ALT (U/L) | 0.413 | 0.200-0.626 | 0.307 |
| AST (U/L) | 0.463 | 0.274-0.652 | 0.662 |
| BUN (mmol/L) | 0.598 | 0.400-0.795 | 0.250 |
| Cr (μmol/L) | 0.629 | 0.459-0.799 | 0.128 |
| UA (μmol/L) | 0.532 | 0.376-0.689 | 0.703 |
| Total cholesterol (mmol/L) | 0.439 | 0.299-0.580 | 0.475 |
| Triglycerides (mmol/L) | 0.393 | 0.233-0.554 | 0.209 |
| HDL (mmol/L) | 0.375 | 0.195-0.555 | 0.141 |
| LDL (mmol/L) | 0.484 | 0.331-0.638 | 0.854 |
| Total T (cells/μL) | 0.447 | 0.271-0.624 | 0.534  0.863  0.633  0.243  0.299  0.332  0.757  0.712  0.939  0.192  0.363  0.599  0.344  0.288  0.272  0.739  0.498  0.238  0.748 |
| T% | 0.485 | 0.316-0.655 |  |
| Total B (cells/μL) | 0.541 | 0.390-0.691 |  |
| B% | 0.599 | 0.438-0.760 |  |
| NK (cells/μL) | 0.412 | 0.247-0.576 |  |
| NK% | 0.418 | 0.254-0.581 |  |
| CD4^+^T cells | 0.474 | 0.301-0.647 |  |
| CD4^+^T% | 0.531 | 0.348-0.715 |  |
| CD8^+^T cells | 0.494 | 0.305-0.682 |  |
| CD8^+^T% | 0.389 | 0.246-0.533 |  |
| Th1 (cells/μL) | 0.423 | 0.257-0.589 |  |
| Th1% | 0.545 | 0.371-0.718 |  |
| Th2 (cells/μL) | 0.58 | 0.419-0.742 |  |
| Th2% | 0.41 | 0.258-0.562 |  |
| Th17 (cells/μL) | 0.407 | 0.233-0.580 |  |
| Th17% | 0.528 | 0.363-0.693 |  |
| Treg (cells/μL) | 0.558 | 0.389-0.726 |  |
| Treg % | 0.6 | 0.431-0.769 |  |
| Th17/Treg | 0.473 | 0.323-0.623 |  |
| IL-2 (pg/ml) | 0.692 | 0.566-0.817 | 0.024* |
| IL-4 (pg/ml) | 0.508 | 0.379-0.638 | 0.923 |
| IL-6 (pg/ml) | 0.538 | 0.398-0.679 | 0.650 |
| IL-10 (pg/ml) | 0.51 | 0.349-0.670 | 0.911 |
| IL-17 (pg/ml) | 0.547 | 0.388-0.706 | 0.577 |
| IFN-γ (pg/ml) | 0.418 | 0.223-0.614 | 0.336 |
| TNF-α (pg/ml) | 0.414 | 0.199-0.630 | 0.312 |

Abbreviations：AUC, area under the curve ROC curve; CI, confidence interval. BMI, body mass index; ESR, erythrocyte sedimentation rate; CRP, C-reactive protein; ALT, alanine transaminase; AST, aspartic transaminase; BUN, blood urea nitrogen; Cr, creatinine; UA, uric acid; LDL, low density lipoprotein; HDL, high density lipoprotein; IL-2,: interleukin-2; IL-4, interleukin-4; IL-6, interleukin-6; IL-10, interleukin-10; IL-17, interleukin-17; INF-γ, interferon-γ; TNF-α, tumor necrosis factor-α. (* ***P*** <0.05, ** ***P*** <0.01, *** ***P*** <0.001)


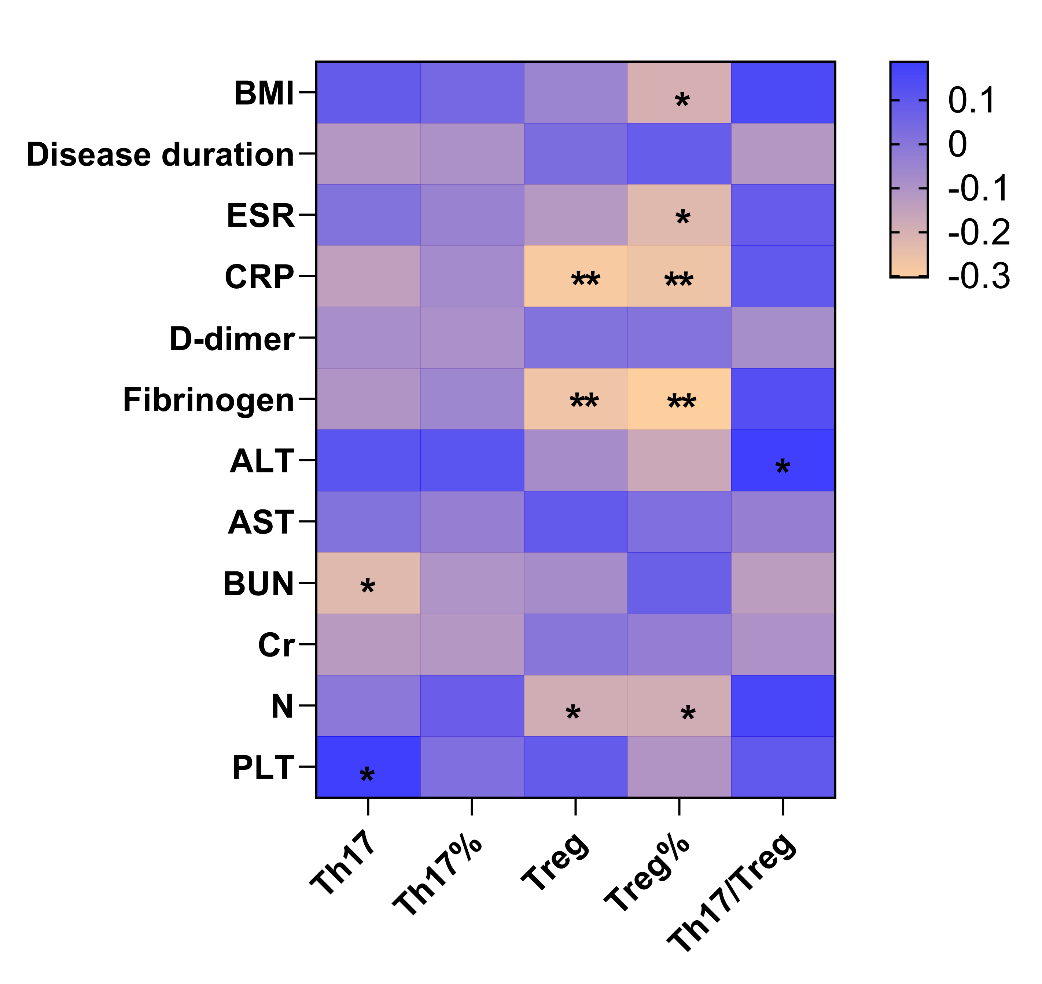


**Supplementary figure S1** Heatmap of correlation of Th17 cells, Treg cells and Th17/Treg ratio with clinical and laboratory characteristics in gout. Th17 cells were positively correlated with PLT, and negatively correlated with BUN. Treg cells had significantly negative correlations with inflammatory indicators and neutrophils. Percentage of Treg cells was negatively correlated with BMI. Th17/Treg ratio was positively correlated with ALT. (∗p < 0.05, ∗∗p < 0.01, and ∗∗∗p < 0.001)
